# Editorial: High performance sports coaching and athlete transition

**DOI:** 10.3389/fspor.2026.1808224

**Published:** 2026-04-14

**Authors:** Luke Jones, Zoë Avner, Neil Boardman

**Affiliations:** 1 Department for Health, University of Bath, Bath, United Kingdom; 2 Faculty of Health - School of Exercise and Nutrition Sciences, Deakin University, Burwood, NSW, Australia; 3 Sport Health and Exercise Science, University of Hull, Kingston upon Hull, United Kingdom

**Keywords:** coach education, coaching, retirement, sport, transition

The transition experiences of high performance athletes have garnered significant attention in sports scholarship over recent times (1). However, despite this healthy corpus of empirical research, there has been limited discussion of how this work might be drawn upon to help the working coach decide how to act in their applied settings in a way that might make a meaningful difference to their athletes' career and post-career life course. Predominantly, athlete transition/retirement experiences have been explored through a sport psychology lens in an effort to support and develop resilience amongst athletes who inevitably face challenging transitions given the nature of a high performance sports career. What remains clear from the findings of athlete transition research is the fact that athletes still struggle with their transitions, whether they be in-career or as athletes move into initial retirement and beyond (2, 3). Consequently, continued exploration is essential to educate, involve, and advise those engaged and enmeshed in these inevitable and often challenging moments in athletes' lives; for example, coaching practitioners and sports support workers (4). This research topic was borne from the observation that more research was needed to determine the types and techniques of support that might ameliorate the so often reported negative implications of athletic transitions. More work was to be done to ascertain answers to two questions: What is it that sports coaches/sports support workers do, or indeed don't do, that affects an athlete's in-career and out-of-career transition experiences? Furthermore, what research informed ethical athlete management strategies might emerge to help working coaches supplement their important coal-face activities?

Socio-cultural researchers have explored the impact of sports cultures upon athlete transition, suggesting that transition experiences should be understood as being intimately connected to the relations of power that govern high performance sports environments (5). Relatedly, in this research topic, we encouraged our contributors to answer the above question by considering the normalized practices, attitudes, and relationships typical to high performance sports settings as foundational to the transition experiences of athletes. The *Frontiers in Sport and Active Living* Research Topic on the research theme “*High Performance Sports Coaching and Athlete Transition”* presents papers that consider the relationship between the dominant practices, attitudes, and relationships adopted by sports coaches and practitioners and the experiences of athletes as they navigate the multiple and varied transitions common to athletic careers. Moreover, through an interrogation of these relationships and practices, our contributing authors have highlighted important aspects of the coaching process that are relevant to the transition experience, allowing the working coach integral insights into how they might react as they navigate their complex roles.

Identifying the specificity of the challenges of transitioning athletes is an essential step to producing recommendations for busy coaches in applied settings. In their original research paper, Stian An Selbekk et al. shed light on the stressors associated with transition from the context of junior to senior high performance female soccer players in Norway. The stressors identified were both in-game and out-of-game but largely circulate around the players' heightened awareness of the likely need to sustain a dual career pathway—as remains a common requirement in many women's soccer cultures around the world. These authors suggest that facilitating arrangements for combining soccer and education is particularly important for their sample—moreover, that the role of the coach as a proactive agent in this facilitation is likely to lead to positive outcomes for the athletes. These authors' findings indicate that coach education might benefit from considering highlighting “out-of-game stressors” as more prominent/relevant considerations for the coach as they traverse their applied roles.

Next, we have a perspective article from Agnew and Pill. The authors place the concept of “athlete-centred” coaching at the forefront of this paper. They demonstrate the importance of expanding perspectives around athlete-centred coaching beyond competitive performance, to include athlete life and career transitions. Not only should the working coach recognise the performance benefits of adopting this approach in their applied settings, but also, they should see how active athlete centred coaching will have positive downstream implications for athlete wellbeing. Drawing upon Griffen et al.'s (6) athlete-centred coaching model and Jowett's (7) model of 3 + 1Cs, Agnew and Pill provide a framework for coaches using an athlete-centred approach to better consider the athlete retirement transition. In doing so, the authors challenge us to broaden our horizons about the scope of athlete-centred coaching for holistic athlete development. This paper provides a foundation for further research and practice around how athlete-centred coaching can act as a positive influence in the long-term development of athletes both during and after their sporting careers.

The last three contributions are informed by the theoretical framing of French philosopher Michel Foucault and overlap in their shared critique of the dominant disciplinary logic of high-performance sport and its potentially problematic effects. First, Øydna et al. problematise the notion of the “committed athlete” by drawing attention to the relational nature of commitment and how it is shaped by and through broader discourses and institutional structures. Drawing on a range of student-athlete vignettes they illustrate both the progressive narrowing of meaning associated with the construct of *commitment* in line with a dominant performance logic, as well as the unequal distribution of possibilities to enact alternative meanings based on talent development system hierarchies. Their analysis invites a critical re-thinking of the limits and possibilities of dual career pathways for expanding possibilities for being a committed athlete. Second, Gerdin and Pringle interrogate the ethical dimensions of coaching in high-performance sport by drawing attention to how coaching practices shape athlete subjectivities both during and after elite sporting careers. They argue that coaching must be reimagined as an ethical, relational and reflexive practice that goes beyond harm reduction to actively support athlete well-being and meaningful, sustainable transitions beyond sport. They explore how Foucault's concept of ethical self-creation can enable coaches to move towards these broader aims by resisting dominant norms and developing care-based coaching approaches that challenge sport's performance-at-all-cost dominant logic. In doing so, their paper offers a contribution to sports coaching research and practice by illuminating how coaches can engage in ethical self-work and systemic transformation, positioning athletes not only as performers but as whole persons capable of living meaningful lives in and beyond sport. Finally, Boardman et al. shed an important critical light on the role of coaches in shaping athletes' experiences of athletic retirement and their capacities to adjust to post-sport life. Drawing on interview data collected with eight coaches working in the context of Olympic sport in the United Kingdom, their Foucauldian analysis illustrates how Olympic coaches' perspectives and experiences of athletic retirement can be linked to the dominant modernist logic of high-performance sport and related disciplinary and normalizing pressures that characterize Olympic level coaching environments. The significance of their paper lies in drawing attention to the underexamined role of key actors (e.g., coaches) in athletes' athletic retirement experiences, thus making a valuable critical contribution to the study of athletic retirement. Taken together, these Foucauldian papers highlight how the intricacies and practices that constitute applied coaching settings might be re-considered by working coaches, not only in an effort to elicit performance gains, but simultaneously as a real-time means of influencing the nature of athletes' transition experiences.
